# Effects of PPARs Agonists on Cardiac Metabolism in Littermate and Cardiomyocyte-Specific PPAR-γ –Knockout (CM-PGKO) Mice

**DOI:** 10.1371/journal.pone.0035999

**Published:** 2012-04-26

**Authors:** Michelangela Barbieri, Clara Di Filippo, Antonietta Esposito, Raffaele Marfella, Maria Rosaria Rizzo, Michele D'Amico, Franca Ferraraccio, Cristina Di Ronza, Sheng Zhong Duan, Richard M. Mortensen, Francesco Rossi, Giuseppe Paolisso

**Affiliations:** 1 Department of Geriatric Medicine and Metabolic Diseases, Second University of Naples, Naples, Italy; 2 Department of Experimental Medicine, Section of Pharmacology L. Donatelli, Second University of Naples, Naples, Italy; 3 Department of Preventive Medicine, Second University of Naples, Naples, Italy; 4 Key Laboratory of Nutrition and Metabolism, Institute for Nutritional Sciences, Shanghai Institutes for Biological Sciences, Chinese Academy of Sciences, Shanghai, China; 5 Metabolism, Endocrinology and Diabetes Division, Departments of Molecular and Integrative Physiology, Pharmacology, and Internal Medicine, University of Michigan Medical School, Ann Arbor, Michigan, United States of America; Ohio State University Medical Center, United States of America

## Abstract

Understanding the molecular regulatory mechanisms controlling for myocardial lipid metabolism is of critical importance for the development of new therapeutic strategies for heart diseases. The role of PPARγ and thiazolidinediones in regulation of myocardial lipid metabolism is controversial. The aim of our study was to assess the role of PPARγ on myocardial lipid metabolism and function and differentiate local/from systemic actions of PPARs agonists using cardiomyocyte-specific PPARγ –knockout (CM-PGKO) mice. To this aim, the effect of PPARγ, PPARγ/PPARα and PPARα agonists on cardiac function, intra-myocyte lipid accumulation and myocardial expression profile of genes and proteins, affecting lipid oxidation, uptake, synthesis, and storage (CD36, CPT1MIIA, AOX, FAS, SREBP1-c and ADPR) was evaluated in cardiomyocyte-specific PPARγ –knockout (CM-PGKO) and littermate control mice undergoing standard and high fat diet (HFD). At baseline, protein levels and mRNA expression of genes involved in lipid uptake, oxidation, synthesis, and accumulation of CM-PGKO mice were not significantly different from those of their littermate controls. At baseline, no difference in myocardial lipid content was found between CM-PGKO and littermate controls. In standard condition, pioglitazone and rosiglitazone do not affect myocardial metabolism while, fenofibrate treatment significantly increased CD36 and CPT1MIIA gene expression. In both CM-PGKO and control mice submitted to HFD, six weeks of treatment with rosiglitazone, fenofibrate and pioglitazone lowered myocardial lipid accumulation shifting myocardial substrate utilization towards greater contribution of glucose. In conclusion, at baseline, PPARγ does not play a crucial role in regulating cardiac metabolism in mice, probably due to its low myocardial expression. PPARs agonists, indirectly protect myocardium from lipotoxic damage likely reducing fatty acids delivery to the heart through the actions on adipose tissue. Nevertheless a direct non- PPARγ mediated mechanism of PPARγ agonist could not be ruled out.

## Introduction

Triglycerides are normally stored in adipocytes while extra adipocyte storage (liver, and myocardium) is minimum and very tightly regulated. However, several-fold increase of triglyceride storage in cardiomyocytes has been observed in animal models of obesity and diabetes [1]–[4] and contributes to cardiomyocyte death by non oxidative and oxidative [5] metabolic pathways. In humans, myocardial lipid content was recently reported to increase with the degree of adiposity and contribute to cardiac dysfunction [6], suggesting myocardial lipid content to be a biomarker and putative therapeutic target for cardiac diseases in patients with Metabolic Syndrome [7].

Understanding the molecular regulatory mechanisms controlling for myocardial lipid metabolism is of critical importance for the development of new therapeutic strategies for cardiac diseases. To this regard, it has been previously demonstrated that interventions leading to reduction in myocardial lipid stores improve cardiac function in rodent models of diabetes [1], [4], [8].

Heart energy is primarily derived from oxidation of FAs, and this correlates with the relatively high levels of PPARα expression in the heart [9]–[10].Activation of these receptors induces expression of a number of genes encoding proteins involved in transmembrane transport and mitochondrial β-oxidation of fatty acids [10]. In contrast, PPARγ is expressed at relatively low levels in the heart [11]–[14] and recent data have shown that tissue specific loss of PPARγ have no effect on expression of gene controlling lipid and glucose metabolism [15]. Furthermore, exposure of myocytes to PPARα or α/γ specific agonists or long-chain fatty acids led to a significant induction of known PPAR target genes involved in fatty acid uptake and oxidation whereas a PPAR agonist had no effect [12].

In animals, thiazolidinediones (TZDs), selective PPARγ activators, are cardioprotective against ischemic insult, reduce myocardial infarct size, ameliorate contractile dysfunction caused by ischemia reperfusion injury [16]–[18] and may modulate cardiac hypertrophic growth response [19]–[21]. In humans, contrasting data have been provided [20]–[27]. PPARγ and thiazolidinediones impact on myocardial lipid metabolism is still debated. In rodent models of lipotoxic dilated cardiomyopathy, PPARγ agonist treatment improved heart function [4], [28]. In contrast, PPAR γ -transgenic mice over-expressing PPAR γ developed a dilated cardiomyopathy associated with increased lipid and glycogen stores and distorted architecture of the mitochondrial inner matrix [29]. In such mice, PPARγ agonists increased mRNA levels of fatty acids (FAs) oxidation and uptake genes and exacerbated heart dysfunction. In contrast, rosiglitazone treatment of wild-type mice reduced expression of PPARγ targets [29].

Due to low myocardial PPARγ expression, in vivo effects of PPARγ agonists on cardiac metabolism are generally thought to be indirect and secondary to their lipid lowering properties [12]. Direct regulation of cardiac metabolism by PPARγ is a subject of considerable debate. Baranowsky et al [13] found that pioglitazone induced lipid accumulation in the heart of rats fed both the standard and high fat diet in spite of concurrent reduction in plasma FFA concentration thus suggesting a direct action of PPARγ agonist on the heart. More recently it has shown that tissue-specific loss of PPARγ alters heart function and induces myocardial hypertrophy [15], [30] with mitochondrial oxidative damage [30] although no effect on gene expression controlling lipid and glucose metabolism at baseline was observed [15]. Indeed, myocardial fatty acids utilization and cardiac contraction were depressed in tamoxifen inducible short term cardiomyocyte restricted PPARγ knockout mice [31].

Interestingly enough, such previous findings suggest the possibility that expression and activity of PPARγ may be induced in the heart only under certain physiological or pathophysiological conditions. Further studies using cardiac-specific PPARγ deletion are required to determine if PPARγ regulation of cardiac gene expression is mediated by direct or indirect mechanisms.

Thus, to better assess the role of PPARγ on cardiac lipid metabolism and myocardial function and differentiate local against systemic actions of PPARγ agonists the effect of PPARγ (rosiglitazone), PPARγ/PPARα (pioglitazone) and PPARα (fenofibrates) agonists on lipid accumulation and cardiac function was evaluated in cardiomyocyte-specific PPARγ knockout (CM-PGKO) mice and littermate control undergoing to standard and high fat diet.

Use of PPARγ –knockout (CM-PGKO) and control mice will allow to: a) compare myocardial lipid metabolism and function in absence and presence of cardiomyocyte PPARγ b) differentiate local from systemic actions of PPARs agonists b) verify if the effect of PPARγ agonist at myocardial level is dependent or independent of cardiomyocyte PPARγ receptor activation.

## Materials and Methods

### Ethics Statement

All the procedures performed on mice conform with the Directive 2010/63/EU of the European Parliament and have been approved from the University Committee on Animal Care and Use at Second University of Naples (Permit Number: 0009352/08/CB). All efforts were made to ameliorate suffering of animals.

### Animals

α MHC-Cre mice (Cre driven by α -myosin heavy–chain [MHC] promoter) [32] (were bred to floxed PPARγ) [33] mice to obtain cardiomyocyte-specific PPARγ knockout (CM-PGKO) mice (homozygous floxed PPARγ and α MHC-Cre positive). CM-PGKO mice were bred to homozygous floxed PPARγ mice to generate both CM-PGKO mice and littermate control mice (homozygous floxed PPARγ and α MHC-Cre negative).

All mice were housed in 12 h light/dark cycle (light on 7 a.m.), temperature 22°C, and given standard chow and water *ad libitum*. After 8 weeks acclimation CM-PGKO and littermate mice were randomly assigned to receive for 18 weeks:

- Group A: high fat diet (HFD) (Purifed diet 230HF-SAFE, AUGY, FRANCE) (60.6% kcal of calories in form of lard fat, 26.3% kcal of calories as carbohydrates 13.1% kcal of calories as protein)
- Group B: standard diet (SD) (3KE28 GLP Basic, MUCEDOLA, MI, ITALY) (containing 13.2% of calories as fat).

After 18 weeks, both groups were randomly assigned to receive the following treatment for 6 weeks:

- Group A1: HFD,
- Group A2: HFD+rosiglitazone (10 mg/Kg/day),
- Group A3:HFD+pioglitazone (20 mg/Kg/day)
- Group A4: HFD+fenofibrate (100 mg/kg/day)
- Group B1: SD
- Group B2: SD+rosiglitazone (10 mg/Kg/day),
- Group B3: SD+pioglitazone (20 mg/Kg/day)
- Group B4: SD+fenofibrate (100 mg/kg/day)

The dose of the drug was chosen according to previous studies in which similar dose was used in mice for investigations of the effect of pioglitazone and rosiglitazone on left ventricular remodelling [21], [34]

### In vivo hemodynamic measurement

Mice were weighed and anesthetized with diazepam (6 mg/kg sc) and Hypnorm™ (0.7 mg/kg fentanyl citrate and 20 mg/kg fluanisone im), and a tracheotomy was performed for artificial ventilation (Harvard Apparatus, AH 40–1000). The tidal volume of the respirator as set at 1 ml/min, with the rate set at 100 strokes/min, and was supplemented with 100% oxygen. Animals were closely monitored during the procedure to assure that they are maintained in the proper anaesthetic plane. In particular, respiration, colour of the mucous membranes, core body temperature and responses to pedal withdrawal reflex stimuli were continuously monitored

Core body temperature was maintained at 37°C with the use of a rectal thermometer attached to a heating pad. The right carotid artery was cannulated with PE-10 tubing, and the mean arterial blood pressure (MABP) was recorded by a computerized data acquisition system (Power Lab Instrument). Heart rate (HR) was recorded during a ECG continuous for 15 s at 50 mm/s velocity. The Pressure rate index (PRI), an index of myocardial oxygen consumption, has been calculated as FC×PAM/1000 and expressed in mmHg/min/10^3^.

### Plasma FFA and glucose levels

Blood from fasted (12 hours) mice was collected and the plasma stored at −20°C until assay. Blood glucose level and FFAs were measured using commercially available enzymatic kits (Wako Chemicals, Richmond, VA and Sigma, St. Louis, MO)

### Cardiac Hypertrophy Estimation

Hearts were removed and ventricles were dissected for weighting. Ventricular weight to body weight ratio (VW/BW) (milligrams per gram) was used as indicators of cardiac size. Left ventricles were then snap-frozen in liquid nitrogen for further analyses.

### Transcript analyses

Total RNA samples were extracted using a RNA extraction kit (Tristapure Eurogetec). QPCR (Quantitative real-time RT-PCR) analyses (QUANTICA sequence detection system-TECHNE) were carried out to determine transcript levels of target genes. The following genes were assayed: PPARγ, PPARα, AOX, CPT1MIIA, CD36, FAS, ADRP, GLUT4 and SREBP1-c. SYBR green was used as a universal fluorescent probe and βACTIN was used as an endogenous control. QPCR results from each gene/primer pair were normalized to βACTIN, and compared across conditions.

In order to validate the efficiency of the target mRNA amplification in relation to the endogenous reference βACTIN mRNA, first, a standard curve was generated with serial dilutions of the cDNA. After amplification, the change in the fluorescence was plotted against the cycle number, and the threshold cycle (CT) was calculated through the Quantica software. Plotting CT values vs log cDNA (ng) revealed efficiencies >96%, and therefore, proofing the suitability of this method for heart samples.

### Protein Analysis

Nuclear and cytosolic proteins were extracted using a protein extraction kit (Proteojet mammalian cell lysis reagent-Fermentas).Samples were subjected to SDS PAGE gels and Immunoblotting was performed. Antibodies were obtained from commercial sources: PPARγ (Cell Signalling 81B8), CD36 (Novus Biological D2712), PPARα(H98 Santa Cruz Sc9000), FAS (C20CG Cell Signalling), SREBP1-c (Novus Biological 100-74542), ACOX1 (H-140 Sc 98495), CPT1MIIA (alpha Diagnostics)

### Immunohistochemical analyses

Immunohistochemical analyses were performed as previously described [35]. Tissues were stained with hematoxylin and eosin (H and E). The sections of heart tissue were incubated with anti-SREBP-1c (sc-367; 1∶100 dilution; Santa Cruz Biotechnology, Inc.), monoclonal anti-PPARγ (sc-6284; Santa Cruz Biotechnology, Inc.), monoclonal anti-CD36 (Novus Biological 110-59724), and anti- CPT1MIIA (sc-65982; Santa Cruz Biotechnology, Inc.) PPARα (NBP1-032288), FAS (Cell Signalling C2065), ACOX1 (10957-1AP), ADPR (NUDT9 Sc133857). A portion of each specimen was also snap-frozen, and sections were stained with oil red O. Oil red O staining was quantified using Molecular Analysis Software.

### Statistical analysis

Statistical analysis was performed using SPSS-12. Continuous variable was expressed as means ± standard deviations and compared by analysis of variance (ANOVA) with Bonferroni's test. Correlations between continuous variable will be calculate by regression coefficient. P less than 0,05 will be considered statistically significant.

## Results

### General features of the experimental animals

Baseline body weight (36±2.5 g versus 35.7±1.7 g; p>0.05) and blood glucose levels (7.78±0.47 mmol/L versus 7.39±0.37 mmol/L; p>0.05) of CM-PGKO mice were not significantly different from those of their littermate controls. The VW/BW of all ages were significantly greater in CM-PGKO than in the littermate controls (at baseline 4.75±0.26 vs 4.29±0.37; p = 0.026).

Along with the experimental period, body weight of both CM-PGKO (−2.1 g; p = 0.785) and littermate controls (−3.5 g; p>0.8) standard chow fed animals, did not change significantly, while Rosiglitazone treatment produced significant weight gain in littermate control (+20.75 g; p = 0.001).

HFD alone resulted in significantly higher weight gain compared to the standard chow fed group in both littermate control (50.3±6 g; p = 0.001) and CM-PGKO mice (49±3 g p = 0.001). In the HFD fed animals six weeks pioglitazone, rosiglitazone and fenofibrate treatment did not produce significant change in body weight.

**Figure 1:**
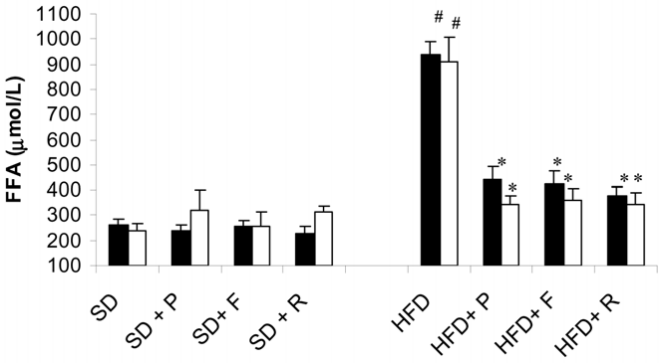
Effect of PPAR γ agonist on FFA plasma levels. Effect of six weeks PPAR agonists treatments on plasma FFA levels of CM-PGKO mice (**white bars**) and littermate controls (**black bars**) fed with standard (SD) or high fat (HFD) diet. # p<0.05 HFD group vs SD group. * HFD +drugs vs HFD group. P = Pioglitazone; F = Fenofibrate; R = Rosiglitazone.

HFD progressively elevated plasma free fatty acids (FFA) concentration in both control (from 290±34 to 940±52 nmol/ml; P<0.05) and CM-PGKO mice (from 309±24 to 912±96 nmol/ml; P<0.05). Six weeks administration of pioglitazone, rosiglitazone and fenofibrate significantly decreased plasma FFA concentration in HFD animals (Fig. 1).

### Cardiac Function

According to previous finding [29], CM-PGKO mice vs littermate control had lower resting heart rate (226±37 vs 320±78; p = 0.034) at baseline. No differences between controls and CM-PGKO mice in PRI (27±4vs23±1 mmHg/min/10^3^; p = 0.168), and mean arterial pressure (116±21vs112±24 p = 0.231) were found even after adjusting for hypertrophy index (p<0.05 for both parameters)

### Expression of PPARs isoforms in the heart

At baseline, the protein levels of PPARγ in littermate control were below the limit of detection with western blot analysis and the percent of positivity at immunoistochimical assay was very low (1%). Also results by quantitative RT-PCR showed low PPARγ mRNA content compared to PPARα ones.

**Figure 2:**
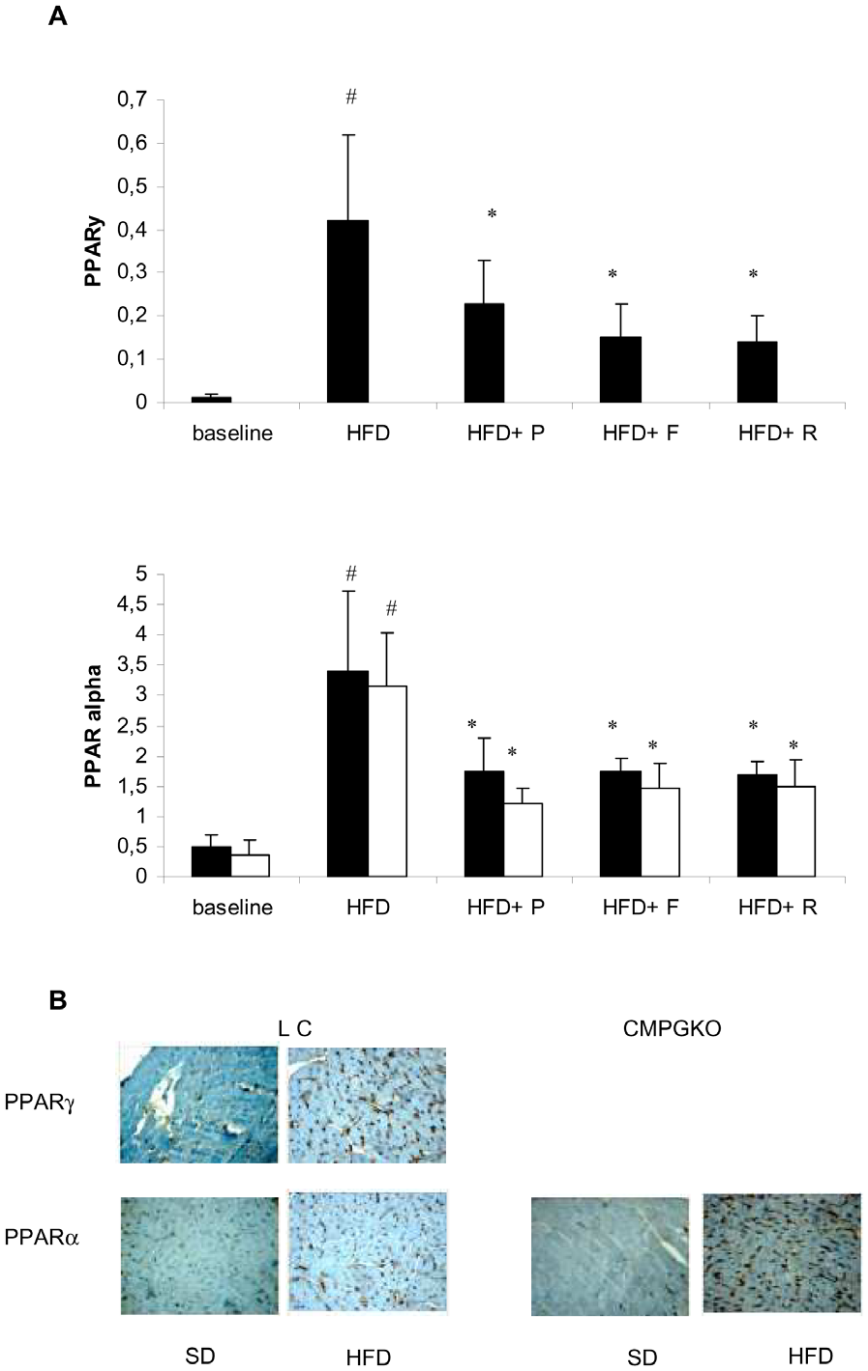
Effect of PPARγ agonist on PPARγ and PPARα mRNA expression and protein levels. (**A**) Effect of six weeks PPAR agonists treatments on PPARγ and PPARα mRNA expression levels in CM-PGKO mice (**white bars**) and littermate controls (**black bars**) at baseline and after 24 weeks high fat (HFD) diet. P = Pioglitazone; F = Fenofibrate; R = Rosiglitazone. # p<0.05 HFD group vs baseline, * HFD +drugs vs HFD group (**B**) Representative immunoistochemical analysis of PPARγ and PPARα protein from ventricular biopsy specimens (×400).

After 24 HFD weeks a progressive increase of PPARγ mRNA expression and protein levels in littermate controls was found (Fig. 2, A and B).

No difference in baseline PPARα gene and protein expression was found between CM-PGKO and littermate controls. HFD increased gene and protein expression of PPARα in both CM-PGKO mice and in littermate controls and induced a progressive increase of PPARγ in littermate controls (fig. 2 A and B).

In HFD fed animals, six weeks administration of pioglitazone, rosiglitazone and fenofibrate significantly decreased PPARγ expression in littermate controls and PPARα in both mice groups (fig. 2 A and B).

### Cardiac Glucose and Lipid Metabolism Gene Expression and Protein Levels

Genes involved in cardiac glucose and lipid metabolism were assessed by quantitative real-time PCR. At baseline, protein levels and mRNA expression of genes involved in lipid uptake, oxidation, synthesis, and accumulation (CD36, CPT1MIIA, AOX, FAS, SREBP1-c and ADPR) of CM-PGKO mice were not significantly different from those of their littermate controls (data not shown). No difference in GLUT4 gene expression and protein levels was also found (data not shown).

**Figure 3:**
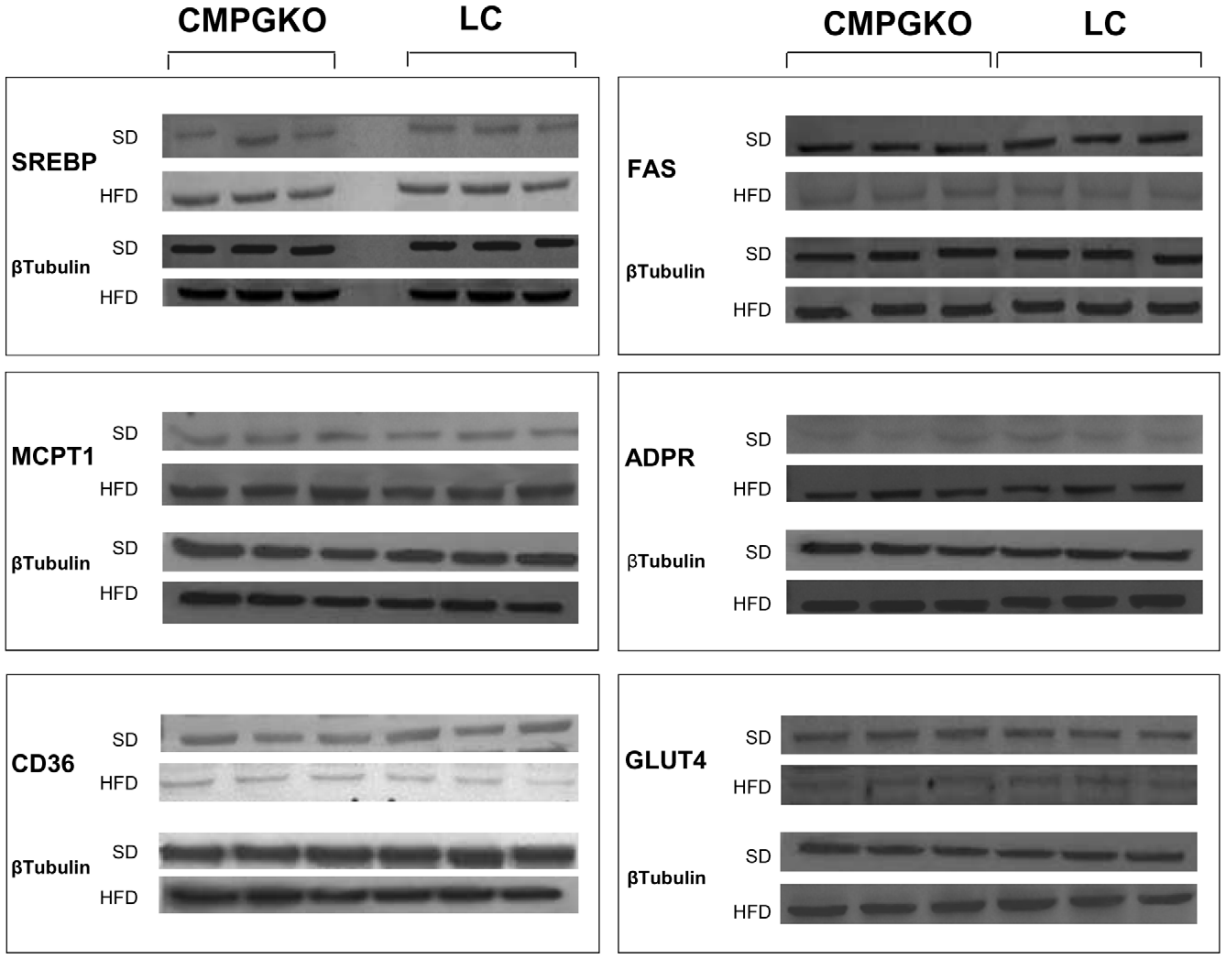
Proteins expression involved in glucose uptake and lipid uptake, oxidation and accumulation. Western blot showing the expression of proteins involved in glucose uptake and lipid uptake, oxidation and accumulation in CM-PGKO and littermate control fed with standard (SD) or high fat diet (HFD).

**Figure 4:**
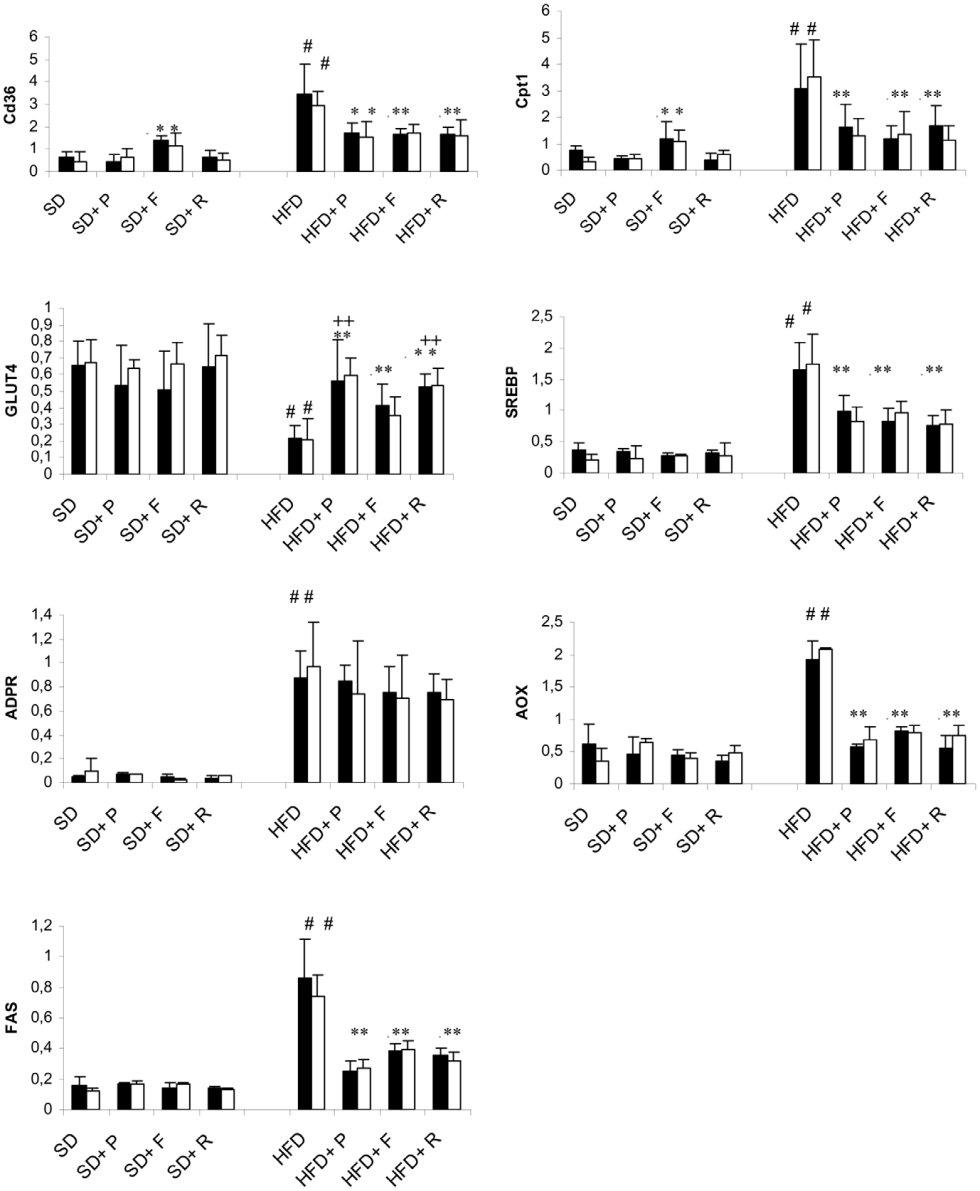
Effect of PPARγ agonist on mRNA expression of genes involved in glucose and lipid metabolism. Effect of Rosiglitazone (R), Pioglitazone (P) and Fenofibrate (F) treatment on cardiomyocyte mRNA expression of genes involved in glucose and lipid metabolism of CM-PGKO mice (**white bars**) and littermate controls (**black bars**) fed with standard (SD) or high fat (HFD) diet for 24 weeks. # p<0.05 HFD group vs SD group. * HFD +drugs vs HFD group. +Piolitazone and Rosiglitazone group vs Fenofibrate.

As expected, 24 weeks high fat diet induced a progressive increase in protein levels and expression of genes involved in lipid uptake, oxidation and accumulation and a decrease in GLUT4 gene expression in both CM-PGKO and littermate controls (Fig. 3 and Fig. 4).

In both CM-PGKO and littermate controls standard chow fed animals, six weeks of fibrates treatment increased CD36 and CPT1MIIA mRNA levels (Fig. 4).

In HFD fed animals, six weeks administration of pioglitazone, rosiglitazone and fenofibrate significantly decreased protein levels and expression of genes involved in lipid uptake, oxidation and accumulation and significantly increased level of cardiac GLUT4 expression in both groups (Fig. 4). In both groups, the increase in GLUT4 expression was significantly higher in pioglitazone and rosiglitazone group compared to fenofibrates one (respectively 1.36 and 1.27 fold in littermate control and 1.6 and 1.49 fold in CM-PGKO). No effect on ADPR mRNA expression was found.

### Heart lipid accumulation

**Figure 5:**
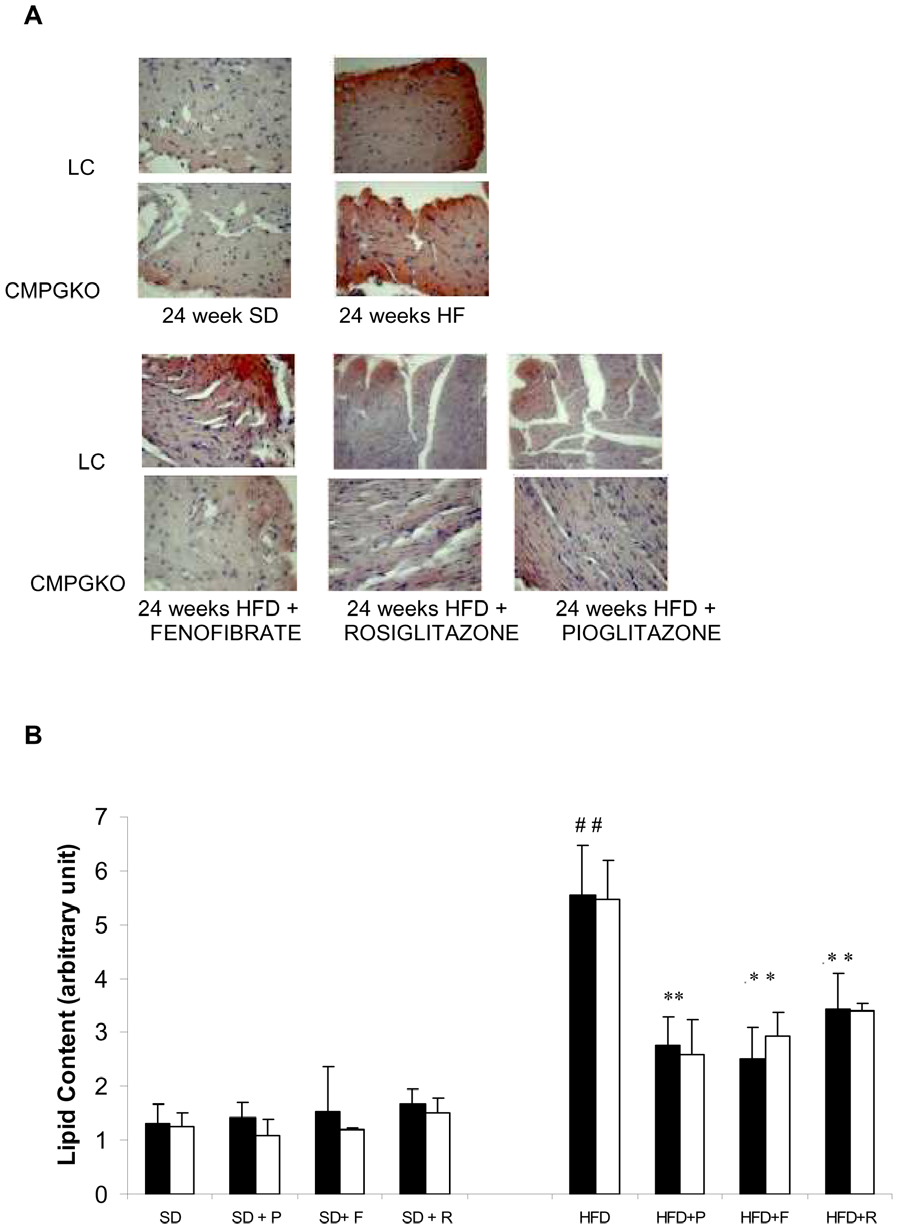
Effect of PPAR agonists on Myocardial lipid content. Effect of PPAR agonists on Myocardial lipid content in CM-PGKO mice (**white bars**) and littermate controls (**black bars**) fed with standard (SD) and high fat diet (HFD). A) Oil red O staining showed an abundance of neutral lipid droplets randomly scattered throughout the cytoplasm of cardiomyocytes (original magnification, ×400). (**B**) Oil red O staining was quantified using Molecular Analysis Software (*n* = 3 in each group). # p<0.05 HFD vs SD, * p<0.05 Drug vs only diet group, +p<0.05 CTLR vs CMPGKO group.

At baseline, no difference in myocardial lipid content was found between CM-PGKO and littermate controls. In both group, twenty four high fat feeding weeks induced a statistically significant progressive increase in the myocardial lipid accumulation. (Fig. 5 A and B)

PPAR agonists did not induced statistically significant change of myocardial lipids in mice fed with SD (Fig. 5 B). On the contrary, in the HFD fed animals six weeks treatment with pioglitazone and rosiglitazone and fenofibrate, induced a significant decrease in lipid content in both CM-PGKO and littermate controls (Fig. 5 A and B)

The myocardial lipid content decrease significantly correlated with plasma FFA decrease (r = 0.68; p = 0.001)

## Discussion

To better assess the role of PPARγ on cardiac lipid metabolism and myocardial function and to better differentiate local/from systemic actions of PPARγ agonists the effect of PPARγ (rosiglitazone), PPARγ/PPARα (pioglitazone) and PPARα (fenofibrates) agonists on lipid accumulation and cardiac function was evaluated in cardiomyocyte-specific PPARγ –knockout (CM-PGKO) mice and littermate control undergoing to standard and high fat diet. Use of PPARγ –knockout (CM-PGKO) and control mice allowed us: a) to compare myocardial lipid metabolism and function in absence and presence of PPARγ; b) to verify if the effect of PPARγ agonist at myocardial level was dependent or independent of cardiomyocyte PPARγ receptor activation.

Our study demonstrate that: a) at baseline, PPARγ does not play a crucial role in regulating cardiac metabolism in mice. When the cardiac FFA afflux increase, (such as in high fat diet, diabetes or metabolic syndrome), myocardial lipid uptake, oxidation and accumulation increase independently of PPARγ receptor activation. Such results, showing a minor effect of PPARγ on myocardial metabolism, do not exclude that PPARγ may have other physiological roles in cardiomyocytes; in fact, different myocardial size/weight and resting heart rate between CM-PGKO mice and littermate control has been found; b) the effect of PPARγ agonist at myocardial level seems to be independent of PPARγ receptors. PPARs agonists, indirectly protect myocardium from lipotoxic damage probably reducing the delivery of fatty acids to the heart via actions on adipose tissue although a direct action in the heart through not PPARγ mediated mechanisms could not be also ruled out.

The role of PPARγ as a key regulator of adipose development and metabolism has been extensively characterized [36]–[37]. However, the exact mechanisms by which PPARγ exerts its effects on cardiac metabolism and function are unclear. Previous studies in mice have generally shown low levels of PPARγ in heart and myocytes and suggested that most of the cardiac effects of PPARγ may be mediated by indirect mechanisms [11]–[14]. However, several recent studies have indicated that tissue-specific loss of PPARγ alters heart function [30] and that cardiac over expression of PPARγ leads to a dilated cardiomyopathy associated with increased lipid and glycogen stores [29].

Our study confirmed low PPARγ mRNA expression and protein levels in standard diet littermate control mice heart while higher levels were observed in HFD mice. High fat diet was associated with a progressive increase in protein levels and expression of genes involved in lipid uptake, oxidation and accumulation and a decrease in glucose uptake in both CM-PGKO and littermate controls.

Interestingly, at baseline and after 24 weeks HFD, any difference in vacuolated myocytes, protein levels and mRNA expression of genes involved in lipid uptake, oxidation, synthesis, and accumulation between CM-PGKO mice and littermate controls was found, despite the marked difference in myocardial PPARγ expression after an HFD. Such results suggest that PPARγ does not play a crucial role in regulating cardiac lipid metabolism in mice and seem to support the hypothesis that in vivo beneficial effects on cardiac metabolism of selective PPARγ activator, thiazolidinediones (TZDs),observed in mice, are indirect and secondary to their lipid lowering properties [27]. Nevertheless, it should be pointed out that rosiglitazone increased myocardial mRNA levels of fatty acids (FAs) oxidation and uptake genes exacerbating heart dysfunction [29] in transgenic mice over expressing cardiac PPARγ and that pioglitazone induced accumulation of myocardial FFA and DAG in the heart of rats fed both the standard and the high-fat diet [13] thus suggesting a direct actions of PPARγ agonist in the heart.

To better differentiate a local against systemic actions of PPARγ agonists the effect of rosiglitazone and pioglitazone agonists on lipid accumulation and cardiac function was evaluated in cardiomyocyte-specific PPARγ –knockout and littermate controls and compared with the effect of fenofibrates a selective PPARα activators. In standard condition we found that pioglitazone and rosiglitazone treatment have no effect on myocardial metabolism while, fenofibrate treatment significantly increases CD36 and CPT1MIIA gene expression, both involved in lipid uptake as also reported by others [9]. Indeed, in both CM-PGKO and control mice undergoing to HFD, six weeks of treatment with rosiglitazone, fenofibrate and pioglitazone was associated with a reduction of myocardial lipid accumulation and a with a shift of myocardial substrate utilization towards greater contribution of glucose in both groups, as evidenced by decreased in fatty acid oxidation and increased GLUT4 mRNA and protein expression. In both groups, the increase in GLUT4 expression was significantly higher in pioglitazone and rosiglitazone group compared to fenofibrates one. Such results indicate the effect on cardiac lipid metabolism to be independent of cardiomyocyte PPARγ. Due to the significant correlation between decrease in myocardial lipid content and fatty acid oxidation and decrease in plasma FFA, we hypothesize that PPARγ agonists may indirectly protect myocardium from lipotoxic damage probably reducing the delivery of fatty acids to the heart. Indeed, a direct action in the heart through not PPARγ mediated mechanisms could not be also ruled out. To this regard, it has been demonstrated that rosiglitazone activates the expression of adiponectin and its receptors in cultured cardiomyocytes [38] and increases adiponectin levels through a direct activation of the adiponectin promoter [39]. Indeed, adiponectin role in modulating glucose and lipid metabolism via activating AMP-activated protein kinase (AMPK) activities is well known [40].

In conclusion, in baseline condition PPARγ does not play a crucial role in regulating cardiac metabolism in mice, likely due to its low myocardial expression. PPARs agonists, indirectly protect myocardium from lipotoxic damage probably reducing fatty acids delivery to the heart through the actions on adipose tissue, although a direct non- PPARγ mediated mechanism of PPARγ agonist could not be fully excluded.
